# Marker-Assisted Improvement of the Elite Maintainer Line of Rice, IR 58025B for Wide Compatibility (*S5*^*n*^) Gene

**DOI:** 10.3389/fpls.2018.01051

**Published:** 2018-07-20

**Authors:** Rahul Priyadarshi, Hari P. S. Arremsetty, Akhilesh K. Singh, Durga Khandekar, Kandasamy Ulaganathan, Vinay Shenoy, Pallavi Sinha, Vikas K. Singh

**Affiliations:** ^1^International Rice Research Institute, South Asia Hub, Hyderabad, India; ^2^Barwale Foundation Research Centre, Hyderabad, India; ^3^Department of Genetics, Osmania University, Hyderabad, India; ^4^ICAR-Indian Institute of Rice Research, Hyderabad, India; ^5^International Crops Research Institute for the Semi-Arid Tropics, Hyderabad, India

**Keywords:** hybrid rice, wide compatibility, IR 58025B, Maintainer line, Marker-assisted backcross breeding

## Abstract

The degree of heterosis in different hybrid rice varieties is reported to be at the highest in *indica*/*japonica* cross combination, however, there is a problem of sterility and semi-sterility in such inter sub specific hybrids. To overcome this problem, it is essential to develop parental lines having wide compatibility (*S5*^*n*^) gene. In this study, a functional marker S5-InDel was used for marker-assisted backcrossing (MABB) to introgress *S5*^*n*^ gene from Dular into the genetic background of a widely grown recurrent parent IR 58025B, a maintainer line of wild-abortive (WA) cytoplasmic male sterile line, IR 58025A. Further, a closely linked marker *nksbadh2* was used for the identification of plants devoid of aroma in backcross population to develop hybrids with no aroma. The stringent phenotypic selection followed by background selection of BC_3_F_4_ identified plants with 94.51–98.90% of the recurrent parent genome recovery of lines carrying *S5*^*n*^ gene. Subsequently, at 10 promising BC_3_F_5_ lines possessing *S5*^*n*^ gene with high yielding and long-slender grain type were validated for their maintainer behavior through test crosses with IR 58025A. Also the improved lines showed significantly improved spikelet fertility performance while crossed with *japonica* and *javanica* testers in comparison to the original recurrent parent. The improved lines developed in the present study, are being converted to CMS lines through marker-assisted backcross breeding to facilitate precise and improved hybrid breeding program in rice.

## Introduction

Rice is the principal food crop for >50% of world's population and is essential to food security. India ranks first with 44 million hectares area in the world for rice cultivation and second in production with 104.92 million tons. Almost 31% of calories of Indian diet is provided by rice. To feed the continuously growing population of 150 billion by 2030 it is pre-requisite to produce ~130 million tons rice (Indiastat, 2015-16). Exploitation of hybrid vigor in hybrid rice breeding technique is one of the possible options for enhancing rice yield and productivity (Virmani, 1996). Being a self-pollinated crop, there is a need to change pollination system and promote natural out-crossing through induction of male sterility to facilitate hybrid rice breeding. The wild-abortive (WA) is the most commonly used cytoplasm for production of hybrid seed in rice. Because of extensive hard work of several eminent scientists from more than two decades, India has been successfully released 102 hybrid rice from public and private sector for commercial cultivation in the country (ICAR-Indian Institute of Rice Research, 2018). Currently, all the released hybrids are inter-varietal (*indica/indica*) in nature, which does not give a much significant gain of yield advantage over high yielding varieties. It was observed that inter-specific crosses have higher yield heterosis than intra-sub-specific crosses, and the level of yield advantage ranked as *indica*/temperate *japonica* > *indica*/tropical *japonica* > temperate *japonica*/tropical *japonica* > *indica*/*indica* > *japonica*/*japonica* (Yuan, 1994). One of the factors associated with inter-specific *indica*/*japonica* crosses is the formation of sterile pollen grains making the plant sterile. Approximately ~50 loci controlling *indica* × *japonica* hybrid sterility and the loci dealing to overcome sterility, wide compatibility (WC) have been identified to date (Ouyang et al., 2009).

The wide compatibility gene, *S5*^*n*^ is known as one of the locus which is reported to enhance the wide compatibility during the crossed made between *indica* and *japonica* lines (Ouyang et al., 2009). *S5*^*n*^ is a neutral allele that produces fertile offspring when crossed either with *indica* (*S5*^*i*^) or *japonica* (*S5*^*j*^) allele. In this way, *S5*^*n*^ adds to gene flow amongst *indica* and *japonica*. It was found that the *S5*^*n*^ allele contains a 136-bp deletion, which eliminates residues at the N-terminal region of aspartic protease which was not in the case of *S5*^*i*^ and *S5*^*j*^. On comparing sequences of *S5*^*i*^ and *S5*^*j*^, there were two SNPs identified in the coding region, located at 1,010 bp [C/A] and 1,604 bp [C/T] downstream of the start codon and a 1-bp addition/deletion inside the five untranslated region (Chen et al., 2008). The wide compatibility trait in Dular and Moroberekan is controlled by a single dominant gene (Vijayakumar and Virmani, 1992). *S5* and *Sa* are found to be a major locus responsible for the female sterility and male sterility in *indica*-*japonica* hybrids respectively (Long et al., 2008; Yang et al., 2012). Recently, Mi et al. (2016) introgressed two wide-compatibility alleles, *S5-n* and *f5-n*, regulating embryo-sac and pollen fertility, respectively in to an elite *indica* restorer line 9311 to develop new plant type hybrids.

The development of commercial varieties/hybrids is difficult by the direct utilization of wide compatibility (*WC*) gene(s) from a donor because it may not carry all the desired agronomic traits. Therefore, we should first introgressed the desired *WC* gene(s) into the suitable genetic background and then use the improve donor line in the breeding program. Transfer of wide compatibilities gene (*S5n*) into hybrid rice parental line is the preliminary stage for the development of sterility free heterotic *indica/japonica* hybrid. The WC genes present either in male sterile or restorer line is determining factor for high spikelet fertility in F_1_ hybrids. To select improved agronomically superior lines carrying trait of interest can be achieved in faster and precise manner using marker-assisted foreground and background selection.

In the present study, we have improved IR 58025B a maintainer line of IR 58025A (CMS line of 90+ commercially released hybrids in India) with *S5*^*n*^, which is highly stable male sterile lines having wild abortive cytoplasm, good combining ability and heterosis with different restores. The improved lines possessing wide compatibility (*S5*^*n*^) gene would be used as better male sterile lines for exploiting heterosis with diverse restorers (including *Japonica*). The CMS line, IR 58025A is derived from the cross between IR 48483A (as a female parent) and PUSA 167-120-3-2 (as male parent) possessing long slender grain type and mild aroma. The donor line, Dular possessing five different wide compatibility (WC) genes and exhibits highest phenotypic variance for wide compatibility trait. It was noted that Dular produces highly fertile hybrids when crossed with *indica* and *japonica* varieties (Pan et al., 1990; Liu et al., 1996; Zhang et al., 1997; Wang et al., 1998). Interestingly, the hybrids developed from IR 58025B has the property of a mild aroma, which is unprefferable in some parts of India like Southern India. Keeping this in view, we have used marker-assisted backcross breeding strategy coupled with precise phenotypic selection and introgressed WC gene into IR 58025B and developed lines that are agronomically superior and are devoid of aroma.

## Materials and methods

### Plant materials and molecular breeding

The plant materials used in the research work included recurrent parent IR 58025B and donor parent Dular possessing wide compatibility (*S5*^*n*^) gene. The overall Marker-assisted backcross breeding (MABB) approach followed a recurrent backcross procedure including three generations of backcross and four generations of selfing, combined with foreground and background selection (Supplementary Figure 1). A cross was made between the recipient IR 58025B and the donor Dular to generate F_1_ seeds. F_1_ plants were backcrossed with recurrent parent IR 58025B to raise BC_3_F_1_ generations. Marker-selected plants, heterozygous for *S5*^*n*^^+−^ and *badh2*^+−^ loci in the BC_3_F_1_ generation were selfed to generate BC_3_F_2_ generations. In the BC_3_F_2_ generation, plants homozygous for *S5*^*n*^^++^/*badh2*^++^ and *S5*^*n*^^++^/*badh2*^−^ were selected with maximum genome recovery of IR58025B through marker-assisted background selection followed by stringent phenotyping. Further, the selected plants were advanced through pedigree-based phenotypic selection to obtain *S5*^*n*^^++^/*badh2*^++^ as well as *S5*^*n*^^++^/*badh2*^−^ near-isogenic lines (NILs).

All the agronomic performance evaluation and molecular marker analysis were conducted at the Barwale Foundation Research Centre, Hyderabad, India located at 17°24′ 22″ N, 78°12′ 40″ E, and an altitude of 536 m above mean sea level during *Wet season* 2011 to *Wet season* 2015.

### DNA isolation, PCR amplification, and electrophoresis

Fresh leaves were collected from 5 to 6 week-old seedlings of parental lines and backcross progenies. Genomic DNA was isolated following the CTAB method (Dellaporta et al., 1983). DNA samples were quantified on 0.8% agarose gel by comparison with 100/200 ng of Lambda uncut DNA. The DNA was diluted in TE buffer making the final concentration of DNA approximately 25 ng/μl before PCR amplification. BIO-RAD MyCycler thermal cycler was used for performing polymerase chain reaction (PCR). The composition of master mix is 25 nanogram (ng) of template DNA, 0.05 millimolar (mM) of deoxyribonucleotides (dNTPs) (Sigma-Aldrich, USA), 5 picomolar (pM) of each forward and reverse primer, 0.5 units of Taq DNA polymerase (Sigma-Aldrich, USA) and 1X PCR reaction buffer containing 10 millimolar (mM) tris(hydroxymethyl)aminomethane (TRIS), pH 8.4, 50 millimolar (mM) potassium chloride (KCl), 1.5 millimolar (mM) magnesium chloride (MgCl2) and 0.01 milligrams/milliliters (mg/ml) gelatin (Sigma-Aldrich, USA) in a total volume of 15 μl. PCR was performed using with initial denaturation at 94°C for 5 min followed by 35 cycles of PCR amplification under the following parameters: 15 s at 94°C, 30 s at 55°C, and 45 s at 72°C, followed by final extension at 72°C for 6 min. The amplified product *S5*^*n*^ tightly linked markers S5-InDel (Sundaram et al., 2010) and was electrophoretically resolved on 1.5% gels in 1x TAE at 100 V for 80–120 min (Sigma-Aldrich, USA). The amplified product of SSR markers used for background selection and functional marker *nksbadh2* (Singh et al., 2011) for *badh2* gene was electrophoretically resolved on 8% non-denaturing polyacrylamide gel (CBS Scientific, USA). The gel was then stained in ethidium bromide solution (1 μg/ml) for 15 min and de-stained with water and observed on a UV Tran illuminator (Bio-Rad, USA).

### Marker-assisted backcross breeding

#### Foreground selection for wide compatibility gene

A marker-assisted backcross breeding program was adopted for targeted introgression of *S5*^*n*^ locus into IR 58025B background. F_1_ progenies are backcrossed till BC_3_ generation, thereafter the plants progenies were advanced through pedigree method. PCR-based STS marker S5-InDel was used to identify the allelic status of *S5*^*n*^ followed by the functional marker for *badh2* for identification of lines devoid of aroma at BC_1_F_1_ and subsequent backcross generations. Together with genotyping we have implemented stringent phenotyping in each backcross population for all the agronomic and grain quality traits. We have forwarded/selected only those plants which possess maximum recurrent parent genome recovery and phenotypic similarity/superiority to the recurrent parent. Therefore, we have found transgressive segregants for most of the traits in the backcross derived lines. The list of markers used for foreground selection (Supplementary Table 1).

#### Background selection for the recurrent parent genome recovery

A total of 486 simple sequence repeats (SSRs) markers, uniformly distributed throughout the 12 rice chromosomes were taken from the “SSR Markers Resource” (http://archive.gramene.org/markers/) were used for the parental polymorphism survey between IR 58025B and Dular. Polymorphic markers between the two parental lines were used for background selection in backcross populations generated from the crosses (Supplementary Table 2). The estimation of the maximum genome recovery of the recurrent parent genome and selection of the best plant based on SSR marker data was carried out using the software program Graphical GenoTypes (GGT) Version 2.0 (Van Berloo, 1999).

#### Grain quality and agronomic performance evaluations

Thirty days old seedlings of parents and the selected families carrying *S5n* gene were transplanted with 20 × 15 cm spacing, in three replication following a randomized complete block design (RCBD). Twelve rows subplot of each progeny with 15 plants/row were planted. The phenotypic data were recorded for days to 50% flowering (DFF), plant height (cm), productive tillers (number), panicle length (cm), number of filled grains/panicle (number), 1000-grain weight (g), spikelet fertility (%) and grain type. The quality parameters were observed as grain size, kernel length before cooking (KLBC), kernel length after cooking (KLAC), kernel breadth before cooking (KBBC), kernel breadth after cooking (KBAC), length/breadth ratio (LBR), elongation ratio (ER), alkali spreading value (ASV) and aroma was estimated as mentioned in Gopalakrishnan et al. (2008).

#### Assessment of wide compatibility (WC) trait

To estimate wide compatibility in the improved lines, each selected line was evaluated for their wide compatibility trait by progeny testing crossed with appropriate testers and by percentage pollen fertility and spikelet fertility analysis. Two independent crossing programmes were made with IR 58025WCB (possessing *S5*^*n*^ gene) and IR 58025B (without *S5*^*n*^ gene) with five *indica* testers (APO, IR36, IR 64, IR72, and Shan Huang Zhan 2), five *japonica* testers (CT9993, Kinmaze, Nipponbare, Tainung 67, and M 201) and five *tropical japonica* testers (Azucena, Banten, Calotoc Moroberekan, and IR68552-55-3-2) respectively to produce around 20-25 F_1_ seeds per cross during *Dry season* 2014-15. At the time of flowering, spikelet samples from each genotype was collected in 70% alcohol, by removing the anthers from four random unopened spikelets and placed on glass slide in 1% I_2_-KI solution. After crushing the anther on glass slide pollen came out and stained in I_2_-KI solution. The pollen samples were then examined under a light microscope at 40X magnification. More than 200 pollen grains per sample were scored for pollen fertility. Pollen fertility (%) of plants was inferring the relative starch contents, calculated as the ratio of the number of stained pollen grains to the total number of counted pollen grains and multiplies by 100 (Virmani et al., 1997). The seed set in each main panicle was counted to calculate the percentage of spikelet fertility as the ratio of the number of filled spikelet in the panicle to the total number of spikelet in the panicle and multiplies by 100 (Singh et al., 2006).

## Results

### Introgression of wide compatibility (*S5^*n*^*) gene into IR 58025B

Parental polymorphism analysis between recurrent parent IR 58025B and donor parents Dular with 486 SSR markers resulted in the identification of 258 polymorphic markers (53.09%) with more than 5 bp differences between the two lines. Out of 258 polymorphic markers, 91 uniformly distributed SSR markers with larger difference in the product size over the 12 rice chromosomes were used for background selection and percent recurrent parent genome recovery analysis. After confirming heterozygosity with S5-InDel marker and, the true F_1_ plants were backcrossed with the recurrent parent IR 58025B to obtain BC_1_F_1_ plants. All the BC_1_F_1_ plants were screened with S5-InDel marker, and the with 417 bp allele in IR 58025B and 281 bp allele in Dular (*S5*^*n*^^++^) were selected. A total of 960 plants were genotyped, of which 470 plants were wide compatibility (*S5*^*n*^^+−^) gene. Out of these 470 plants, 230 plants were found to be aroma (*badh2*^+−^) gene using *nksbadh2* marker. The gene- based marker *nksbadh2* showed 82 bp in IR 58025B possessing aroma (*badh2*^++^) gene and 90 bp in Dular with the absence of aroma (*badh2*^−^) gene (Supplementary Figure 2). The BC_1_F_1_ plants showed *S5*^*n*^^+−^ and *badh2*^+−^ were also observed for their phenotypic similarity with the IR 58025B (the recurrent parent) to maintain the other agronomically important traits. For background genome recovery information of 14 selected BC_1_F_1_ plant progenies, a total 91 SSR markers were used. The percentage recurrent parent genome (RPG) recovery among the fourteen selected BC_1_F_1_ plants ranged from (70.32 to 74.72%). The best BC_1_F_1_ plant showed maximum recurrent genome recovery of 74.72% and was further selected for BC_2_F_1_ generation.

In BC_2_F_1_ generation, a total of 250 plants were genotyped for *S5*^*n*^ gene, of which 126 plants possessing *S5*^*n*^^+−^ were identified. Out of these 126 plants, 60 were found to be aroma (*badh2*^+−^) gene. However, by agronomic and phenotypic evaluation, 14 plants possessing *S5*^*n*^^+−^ and *badh2*^+−^ were selected for background selection. Using 46 polymorphic SSRs, the percentage of RPG recovery ranged from 80.21 to 86.81%. The best BC_2_F_1_ plant, showed recovery of 86.81% was used for generation of BC_3_F_1._ On the basis of phenotypic data and background selection, four best plants (*S5*^*n*^^+−^ and *badh2*^+−^) having RPG recovery of 92.30%, 92.10, 91.10, and 90.40%, respectively were selfed to generate BC_3_F_2_ generation. In BC_3_F_2_ plants were also screened for *S5*^*n*^ and *badh2* genes to identify homozygous plants. Out of the 800 BC_3_F_2_ plants, 184 plants were *S5*^*n*^^++^ gene. Of these 184 plants, only 46 plants were homozygous for *S5*^*n*^ and devoid of *badh2* genes (*S5*^*n*^^++^/ *badh2*^−^). Additionally, 52 plants were homozygous for *S5*^*n*^ and *badh2* genes (*S5*^*n*^^++^/*badh2*^++^). Ten promising homozygous lines at BC_3_F_5_ generation were identified possessing desired grain type with agronomic trait similar to IR 58025B. Out of these 10 selected families, one family (IR 58025WCB−4-14-5-26-3-8-7) possessing the presence of homozygous for *S5*^*n*^ and *badh2* genes (*S5*^*n*^^++^/*badh2*^++^) and rest nine families possessing homozygous for *S5*^*n*^ and devoid of *badh2* genes (*S5*^*n*^^++^/*badh2*^−^) was supported by phenotype of aroma and wide compatibility trait.

**Figure 1:**
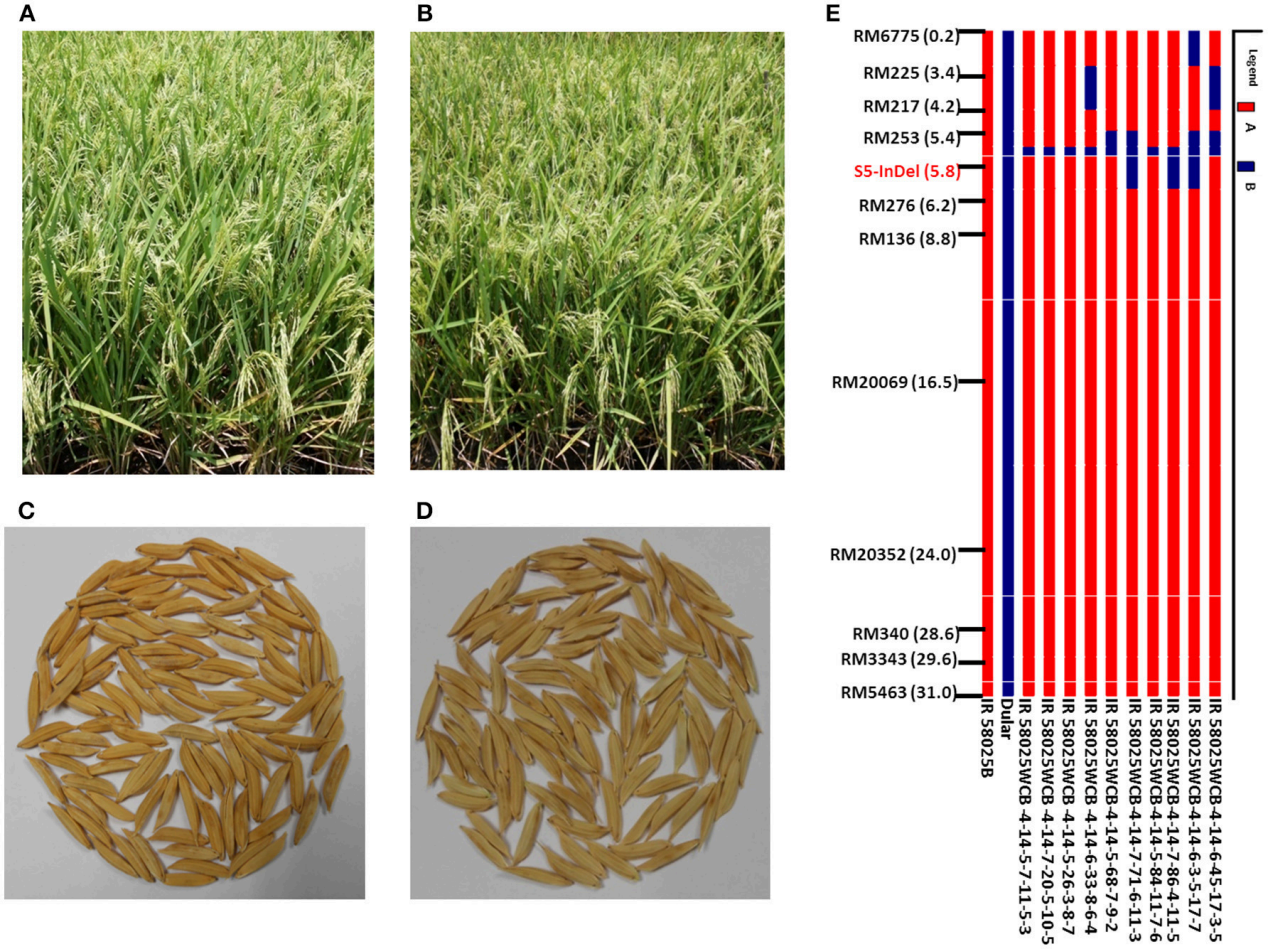
Field view of **(A)** IR 58025B and **(B)** improved IR 58025WCB possessing S5n gene; Grain of **(C)** IR 58025B and **(D)** IR 58025WCB; **(E)** Graphical genotypes of selected BC_3_F_4_ lines with respect to markers on *S5*^*n*^ carrier chromosome 6. Among the 10 improved back cross derived lines, Line No. 3, 4, and 5 (IR 58025WCB−4-14-5-7-11-5-3, IR 58025WCB−4-14-7-20-5-10-5, and IR 58025WCB-4-14-5-26-3-8-7) has minimum linkage drag with maximum recurrent parent genome recovery.

The genome-wide graphical genotype of the *S5*^*n*^ carrier chromosome 6, field view and grain of improved family (IR 58025WCB-4-14-5-7-11-5-3) possessing *S5*^*n*^ along with original parent was presented (Figure 1). The selected families were further subjected for agronomic and cooking quality trait evaluation in comparison to the recurrent parent. The total number of plants generated in each backcross generation and the percentage RPG recovery at each generation is given in Supplementary Table 3.

### Assessment of improved lines for maintainer ability and wide compatibility

#### Maintainer ability

Using the criterion of percentage pollen fertility and percentage spikelet fertility, the maintainer ability of the F_1_s derived from the cross between the selected 10 families and IR 58025A was assessed. It was observed that all the F_1_s had 0% of pollen fertility and percentage spikelet fertility. As a result, it was confirmed the selected ten families displayed perfect maintainer behavior.

#### Wide compatibility

**Figure 2:**
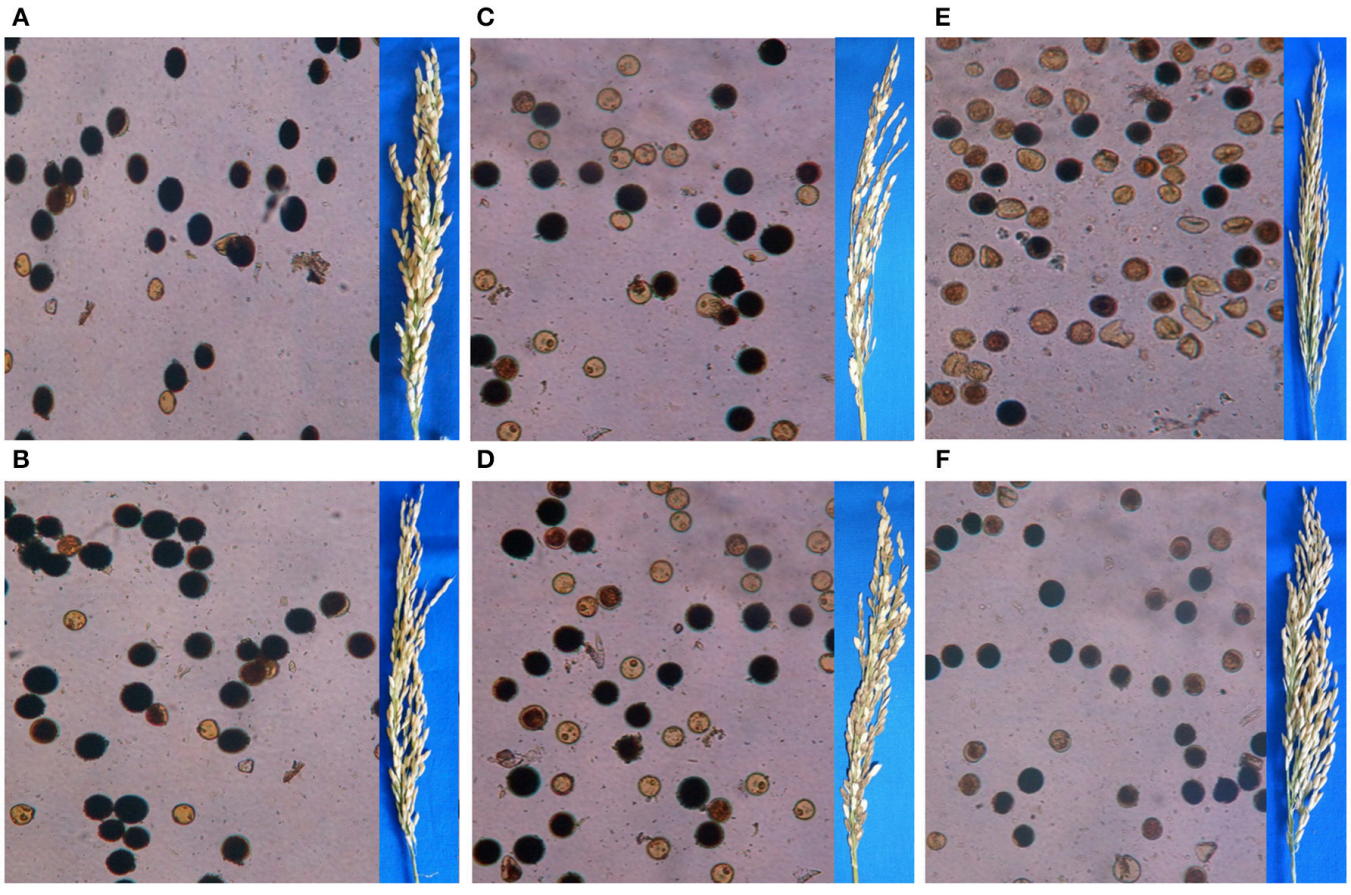
Pollen and spikelet fertility in F_1_ of **(A)** IR 58025B/IR 36; **(B)** IR 58025WCB/IR 36; **(C)** IR 58025B/Tainung 67; **(D)** IR 58025WCB/Tainung 67; **(E)** IR 58025B/ IR 68552-55-3-2; **(F)** IR 58025WCB/IR 68552-55-3-2, respectively.

Crossing of IR 58025B with the 5 *indica*, 5 *japonica*, and 5 *tropical japonica* testers produced F_1_s with pollen fertility ranged from 75 to 85, 35 to 93, and 25 to 68% and spikelet fertility ranged from 75 to 86, 29 to 62, and 51 to 70%, respectively. A significant improvement was observed in spikelet fertility percentage while crossing the improved maintainer line with *S5*^*n*^ gene (IR 58025WCB-4-14-5-7-11-5-3) with the *japonica* and *tropical japonica* testers but the similar result with *indica* testers (Table 1). The pollen and spikelet fertility of F_1_s while using improved maintainer lines ranged from 73 to 88, 60 to 88, and 37 to 82%, and 78 to 88, 68 to 86, and 68 to 73%, for *indica, japonica* and *tropical japonica* testers, respectively. The estimated groups mean pollen percentage increase while using improved maintainer lines with *indica, japonica* and *tropical japonica* testers were 3, 13, and 36% respectively; and for spikelet fertility percentage increase 2, 77, and 15% respectively. Pollen and spikelet fertility in F_1_ with *indica, japonica* and *tropical japonica* testers are presented in Figure 2.

**Table 1:** The comparison of percentage pollen fertility (% PF) and spikelet fertility (% SF) in F_1_s of crosses involving IR 58025B and IR 58025WCB possessing *S5*^*n*^ gene with *different sub-species of rice*.

**MEAN POLLEN AND SPIKELET FERTILITY**									
**Genotypes**	***Indica testers***	**% PF**	**% SF**	***Japonica testers***	**% PF**	**% SF**	***Tropical Japonica testers***	**% PF**	**% SF**
IR 58025B	APO	78	75	Kinmaze	35	29	Moroberekan	38	58
	IR36	82	83	Nipponbare	82	62	IR68552-55-3-2	40	68
	IR72	85	86	Tainung 67	62	43	Azucena	51	53
	IR 64	80	83	CT9993	63	39	Banten	25	51
	Shan Huang Zhan 2	75	82	M 201	93	46	Calotoc	68	70
	Group mean	80	82	Group mean	67	44	Group mean	45	60
IR 58025WCB	APO	85	78	Kinmaze	73	68	Moroberekan	82	68
	IR36	87	84	Nipponbare	85	86	IR68552-55-3-2	55	68
	IR72	88	88	Tainung 67	60	78	Azucena	58	68
	IR 64	73	81	CT9993	75	84	Banten	37	69
	Shan Huang Zhan 2	77	86	M 201	88	73	Calotoc	73	73
	Group mean	82	84	Group mean	76	78	Group mean	61	69

*% PF, Percentage pollen fertility; % SF, Percentage spikelet fertility*.

#### Assessment of improved maintainer lines for agronomic trait

Comparative analysis of backcross-derived improved lines with the recurrent parent showed on part performance for the majority of the traits (Table 2). The date of 50% flowering (DFF) of the improved lines ranged from 92 to 95 days with an average of 94 days similar to that of IR 58025B. The plant height among the families ranged from 93.54 to 95.88 cm in comparison to 93.21 cm of IR58025B. All the selected families showed a significant number of higher effective tillers/plant (13.33–15.17) and filled grain per panicle ranged and from 180.50 to 205.17 in comparison to 13.40 effective tillers/plant and 167.20 filled grain per panicle in IR58025B, respectively. Panicle length among selected families ranged from 25.56 to 26.68 cm in comparison to 25.32 cm in IR58025B. Spikelet fertility (> 75.46%) and test weight (> 20.16 g) of the derived lines was higher in comparison to 74.72% of spikelet fertility and 18.50 g of test weight in IR58025B. The yield advantage of improved lines ranged from 0.19 % to 7.24 % over recurrent parent. Comparative analysis of the datasets of backcross derived lines was helpful to find out the transgressive segregant for plant height, number of tillers, panicle length, filled grains /panicle, percentage spikelet fertility, 1,000 grain weight and yield (q/ha) (Table 2).

**Table 2:** Agronomic performance of the improved IR 58025WCB lines possessing wide compatibility trait.

**Designation**	**DFF (days)**	**PH (cm)**	**NT**	**PL (cm)**	**FG/P**	**SF (%)**	**TW (g)**	**Yield (q/ha)**	**% Sup**	**% RPG**
IR 58025WCB−4-14-5-7-11-5-3	94 ± 1.00	95.24 ± 2.10^*^	14.07 ± 0.90	25.90 ± 0.50	182.10 ± 5.29^*^	75.72 ± 0.68	20.81 ± 0.61^*^	51.20 ± 0.57^*^	3.97	98.90
IR 58025WCB−4-14-7-20-5-10-5	94 ± 0.57	95.88 ± 2.02^*^	14.17 ± 0.05	26.61 ± 0.57^*^	199.10 ± 2.66^*^	75.46 ± 0.48	20.73 ± 0.43^*^	50.41 ± 2.08	2.36	98.90
IR 58025WCB−4-14-5-26-3-8-7	95 ± 0.57	95.23 ± 0.92^*^	15.17 ± 1.00^*^	25.56 ± 0.48	180.50 ± 3.76^*^	80.35 ± 0.66^*^	21.42 ± 0.63^*^	50.69 ± 2.08	2.93	98.90
IR 58025WCB−4-14-6-33-8-6-4	92 ± 1.52	94.87 ± 2.03^*^	13.87 ± 0.49	26.02 ± 0.36	185.47 ± 6.09^*^	77.27 ± 0.63^*^	22.33 ± 0.91^*^	49.34 ± 2.51	0.19	98.90
IR 58025WCB−4-14-5-68-7-9-2	92 ± 2.08	94.92 ± 2.07^*^	14.50 ± 1.03	26.68 ± 0.75^*^	194.43 ± 2.08^*^	79.37 ± 0.65^*^	20.54 ± 0.39^*^	50.03 ± 2.08	1.58	97.80
IR 58025WCB−4-14-7-71-6-11-3	93 ± 1.52	94.18 ± 1.68	13.90 ± 0.60	26.17 ± 0.82	205.17 ± 2.95^*^	79.02 ± 0.36^*^	21.68 ± 0.76^*^	49.64 ± 2.08	0.80	97.80
IR 58025WCB−4-14-5-84-11-7-6	94 ± 1.15	93.54 ± 1.48^*^	14.07 ± 0.80	26.28 ± 0.17^*^	201.20 ± 3.46^*^	79.18 ± 0.85^*^	20.16 ± 0.06^*^	50.26 ± 1.52	2.04	97.80
IR 58025WCB−4-14-7-86-4-11-5	93 ± 1.52	94.21 ± 1.99	14.20 ± 0.88	26.52 ± 0.69^*^	188.43 ± 2.55^*^	76.28 ± 1.77	21.39 ± 0.64^*^	51.39 ± 3.00^*^	4.35	96.70
IR 58025WCB−4-14-6-3-5-17-7	95 ± 1.00	94.85 ± 2.05^*^	13.33 ± 1.66	26.00 ± 0.49	188.83 ± 4.12^*^	82.34 ± 1.91^*^	21.84 ± 0.48^*^	51.11 ± 2.08^*^	3.78	95.60
IR 58025WCB−4-14-6-45-17-3-5	95 ± 1.15	94.84 ± 2.01^*^	14.13 ± 0.05	25.98 ± 0.58	197.13 ± 1.67^*^	79.84 ± 0.47^*^	20.77 ± 0.49^*^	52.82 ± 1.15^*^	7.24	94.51
IR 58025B	94 ± 1.00	93.21 ± 0.05	13.40 ± 0.09	25.32 ± 0.34	167.20 ± 0.95	74.72 ± 0.10	18.50 ± 0.15	49.25 ± 0.25		
CD(0.05)	2.08	1.06	1.27	0.85	6.47	1.56	0.79	1.78		

*DFF, Days to 50% flowering; PH, Plant height; NT, No. of tillers; PL, Panicle length; FG/P, Filled grains /panicle; % SF, Percentage spikelet fertility; TW, 1,000 grain weight; % Sup, percent superiority over recurrent parent (IR 58025B); % RPG, Percent recovery of recurrent parent genome; ±, Standard deviation; CD, Critical difference*;

* *Indicates significant difference from recurrent parent IR 58025B at 5% probability*.

#### Grain and cooking quality of IR 58025WCB lines

The kernel breadth before cooking (KLBC) and kernel length after cooking (KLAC) of the selected ten improved families ranged from 7.03 to 7.32 mm in comparison to 6.96 mm in IR 58025B and 7.46 to 8.65 mm in comparison to 8.15 mm of IR 58025B, respectively (Table 3). All the selected families showed same alkali spreading value (ASV) score of 6 with that of the recurrent parent, IR58025B. The family (IR 58025WCB -4-14-5-26-3-8-7) showed similar aroma (score of 1) like IR58025B, while rest nine families showed the absence of aroma (score of 0). The elongation ratio in the improved lines ranged from 1.04 to 1.20 in comparison to 1.17 in IR 58025B and 1.18 of Dular.

**Table 3:** Grain and cooking quality traits of the improved IR 58025WCB lines possessing wide compatibility rait.

**Designation**	**Grain shape**	**KLBC (mm)**	**KBBC (mm)**	**L/B**	**KLAC (mm)**	**KBAC (mm)**	**ER**	**ASV**	**Aroma**
IR 58025WCB−4-14-5-7-11-5-3	Long	7.31 ± 0.15^*^	1.77 ± 0.01^*^	4.14 ± 0.07	8.56 ± 0.27	2.05 ± 0.08	1.17 ± 0.05	6	0
IR 58025WCB−4-14-7-20-5-10-5	Long	7.26 ± 0.20^*^	1.72 ± 0.03	4.23 ± 0.17	8.40 ± 0.15	1.94 ± 0.03	1.16 ± 0.05	6	0
IR 58025WCB−4-14-5-26-3-8-7	Long	7.24 ± 0.16^*^	1.75 ± 0.03^*^	4.15 ± 0.04	8.51 ± 0.16	1.98 ± 0.04	1.18 ± 0.01	6	1
IR 58025WCB−4-14-6-33-8-6-4	Long	7.06 ± 0.12	1.72 ± 0.01	4.11 ± 0.05	8.32 ± 0.41	2.04 ± 0.08	1.18 ± 0.07	6	0
IR 58025WCB−4-14-5-68-7-9-2	Long	7.05 ± 0.15	1.75 ± 0.02^*^	4.03 ± 0.11	8.23 ± 0.05	1.88 ± 0.01^*^	1.17 ± 0.03	6	0
IR 58025WCB−4-14-7-71-6-11-3	Long	7.23 ± 0.24^*^	1.71 ± 0.01	4.24 ± 0.13	8.65 ± 0.29	1.86 ± 0.01^*^	1.20 ± 0.02	6	0
IR 58025WCB−4-14-5-84-11-7-6	Long	7.03 ± 0.17	1.81 ± 0.02^*^	3.89 ± 0.13	7.46 ± 0.15^*^	1.87 ± 0.05^*^	1.06 ± 0.04^*^	6	0
IR 58025WCB−4-14-7-86-4-11-5	Long	7.32 ± 0.19^*^	1.77 ± 0.05^*^	4.13 ± 0.15	7.62 ± 0.13	1.84 ± 0.04^*^	1.04 ± 0.01^*^	6	0
IR 58025WCB−4-14-6-3-5-17-7	Long	7.30 ± 0.06^*^	1.74 ± 0.02^*^	4.20 ± 0.04	8.10 ± 0.04	1.84 ± 0.01^*^	1.11 ± 0.01	6	0
IR 58025WCB−4-14-6-45-17-3-5	Long	7.12 ± 0.17	1.74 ± 0.07^*^	4.08 ± 0.10	8.04 ± 0.01	1.87 ± 0.05^*^	1.13 ± 0.02	6	0
Dular	Medium	5.97 ± 0.05	1.93 ± 0.02	3.09 ± 0.05	7.02 ± 0.32	1.94 ± 0.08	1.18 ± 0.06	4	0
IR 58025B	Long	6.96 ± 0.26	1.68 ± 0.02	4.15 ± 0.15	8.15 ± 0.01	2.05 ± 0.01	1.17 ± 0.04	6	1
CD(0.05)		0.21	0.05	0.17	0.60	0.14	0.08		

*Grain shape: (Extra Long: > 7.50mm, Long: 6.61–7.50 mm, Medium: 5.51–6.60, Short <5.00 mm); KLBC: Kernel length before cooking; KBBC, Kernel breadth before cooking; L/B ratio, Length/Breadth ratio; KLAC, Kernel length after cooking; KBAC, Kernel breadth after cooking; ER, Elongation ratio; ASV, Alkali spreading value (1–2 high and 6–7 Low); Aroma, (0 absent, 1 mild, 2 strong and 3 very strong); ±, Standard deviation; CD, Critical difference*;

* *Indicates significant difference from recurrent parent IR 58025B at 5% probability*.

## Discussion

The major objective of the present study is to introgress a wide compatibility gene, *S5*^*n*^ into the background of a WA cytoplasm containing maintainer line, IR 58025B through marker-assisted backcross breeding coupled with rigorous phenotypic observation for improved agronomic traits and grain characteristics. The maintainer line used in the present study is a parent of more than 90% of hybrids released in India for commercial cultivations. Improvement of this line for wide compatible (WC) trait facilitate the development of new generation of hybrids with higher heterosis. Sundaram et al., (2010) have developed *S5*^*n*^ gene-based functional marker (S5-InDel), which paved the path to transfer the alleles in any recurrent parent. Foreground selection of *S5*^*n*^ gene was performed using S5-InDel marker, at seedling stage and very stringent phenotypic observation in favor of the recurrent parent phenotype especially yield and its related components, and grain characteristics followed by background analysis to develop and identify the improved version IR 58025WCB possessing *S5*^*n*^ gene.

During agronomic trait evaluation, it was found that the developed advanced breeding lines of IR 58025WCB had similar days to 50% flowering data. The higher yield performance of the improved IR 58025WCB families was primarily observed due to an increase in panicle length, filled grain per panicle, grain yield, and test weight. However, the improved IR 58025B lines with *S5*^*n*^ gene showed higher percent spikelet fertility, indicating no linkage drag effect while transferring the *S5*^*n*^ gene locus. The L/B ratio of all improved lines possessing *S5*^*n*^ gene is not statistically significant as compared to IR 58025B. Out of ten, two improved families possessing *S5*^*n*^ gene showed elongation ratio different and statistically significant and eight improved families showed not statistically significant, as compare to IR 58025B. Out of ten, nine improved families possessing *S5*^*n*^ gene showed the absence of aroma trait and one improved family showed the presence of aroma trait. The hybrids developed using IR 58025A (CMS lines) possess mild aroma, a trait which is not preferred in many parts of India, particularly in South India. The negative selection of aroma trait was done through eliminating the aroma alleles during MABB. We were able to identify plants that do not possess aroma (i.e., plants that are the absence of aroma trait with respect to the major gene for fragrance, *badh2*) at each generation by using a functional marker for aroma trait, viz. *nksbadh2* marker (Singh et al., 2011). Thus, the selection for the negative alleles for *badh2* resulted in the identification of plants which is devoid of aroma in the improved maintainer line possessing wide compatibility trait.

Ninety-one polymorphic SSR markers were used across the genome, distributed at an average interval of 4.27 Mb were employed to analyze the recovery of recurrent parent genome (RPG) in the IR 58025WCB families. The percentage recurrent parent genome recovery of the selected 10 introgression lines ranged from 94.51 to 98.90%. It was due to stringent phenotypic and background selection performed at each backcross generation. Further, foreground and background selections coupled with strict phenotypic observation were used to recover the recurrent parent genome (RPG) up to 97.3% in two backcross generations (Basavaraj et al., 2010). The high level of heterosis is limited in the cross of *indica* and *japonica* genotypes due to hybrid sterility in F_1_. Improvement of IR 58025B with *S5*^*n*^ gene overcome embrosac sterility, when crossed with the *japonica* testers. These lines showed improvement in both pollen and percent spikelet fertility in F_1_s, which is generated from *japonica* and *tropical japonica* testers. But it was observed that, the percent spikelet fertility almost similar with the *indica* testers.

The cause of different level of expression in different background is epistasis or non-allelic interactions (Kubo and Yoshimura, 2000; Kubo et al., 2008). A different degree of expression of *WC* genes suggested the presence of modifier gene(s) and epistasis (Kumar and Chakrabarti, 2000). Wide compatible varieties (WCVs), when used as male parents exhibited positive effect regarding a number of spikelets/panicle and percentage spikelet fertility in their hybrids with *indica* and *japonica* testers (Vijaya Kumar et al., 1999). We have found higher spikelet fertility while crossing the improved maintainer lines with *japonica* and *javanica* testers. The derived lines possessing wide compatibility traits will be useful in overcoming the problem of intersubspecific hybrid sterility and exploitation of the strong *indica*-*japonica* heterosis (Guo et al., 2016). The lines possessing *S5*^*n*^ was improved by 14.7–32.9% embryo-sac fertility in *indica-japonica* hybrids. (Mi et al., 2016). The improved lines showed similar maintainer, like IR 58025B when crossed with IR 58025A (WA-CMS line).

Based on the several reports it is clear that the commercially released hybrids (100+) available in India is *indica* (CMS and mostly IR58025A*)-indica* (many restorers) in nature. Further, development of high yielding next generation hybrids development of *indica-japonica* heterosis is one of the available options. Therefore, instead to improve the restorer line(s) (available large in number) with *S5*^*n*^, we have improved IR 58025A (CMS line of 90+ hybrids) which is highly stable having wild abortive cytoplasm, good combining ability and heterosis with different restores. Many released hybrids in India like KRH2, Sahyadri and DRRH1 by public sector and 6444 by private sector is using IR 58025A as one of the parent (ICAR-Indian Institute of Rice Research, 2018). The improved lines may use as donor for wide compatibility trait in different background to improve *indica*-*japonica* better heterosis.

## Conclusion

In the present study, *S5*^*n*^ gene was introgressed in the IR 58025B and *badh2* gene-based functional marker *nksbadh2* used for the identification of improved lines with *S5*^*n*^ gene and devoid of aroma trait by MABB. The IR 58025WCB possessing *S5*^*n*^ gene was either similar or better in agronomic traits performance and grain characteristics compared to recurrent parent IR58025B. This work has offered an example for the combination of WC trait with and without aroma by MAS especially for the future development of WC *japonica* sterile line, although the possible range of the breeding application of *indica* sterile line with *S5*^*n*^ may be largely limited by the pollinating characters of the corresponding *japonica* restorer for an inter-subspecific cross. The improved line may use for better exploitation of heterosis using *indica*/*japonica* derivative restorers. The development of new plant type and super rice breeding can be possible by combining desirable characters of *indica* genotypes such as grain shape, grain quality, plant texture, resistance to pest, with desirable characters of *japonica* genotypes such as lodging resistance, cold tolerance, high photosynthetic efficiency and early maturity and reciprocally through combination breeding into maintainers.

## Author contributions

RP, VS, and HA was involved in the design of the experiment. RP conducted the experiment, analyzed the data and wrote the manuscript; AS, DK, and KU helped in experimental work, and contributed to the manuscript modification. PS and VKS was involved in revising the manuscript. All authors approved the final version of the manuscript.

### Conflict of interest statement

The authors declare that the research was conducted in the absence of any commercial or financial relationships that could be construed as a potential conflict of interest.

## References

[B1] Basavaraj S. H. Singh V. K. Singh A. Singh A. Singh A. Yadav S. (2010). Marker-assisted improvement of bacterial blight resistance in parental lines of Pusa RH10, a superfine grain aromatic rice hybrid. Mol. Breed. 26, 293–305. 10.1007/s11032-010-9407-3

[B2] Chen J. Ding J. Ouyang Y. Du H. Yang J. Cheng K.. (2008). A triallelic system of *S5* is a major regulator of the reproductive barrier and compatibility of *indica-japonica* hybrids in rice. Proc. Nat. Acad. Sci. USA 105, 11436–11441. 10.1073/pnas.0804761105 18678896 PMC2516230

[B3] Dellaporta S. L. Wood J. Hicks J. B. (1983). A plant DNA mini preparation: version II. Plant Mol. Biol. Rep. 1, 19–21. 10.1007/BF02712670

[B4] Gopalakrishnan S. Sharma R. K. AnandRajkumar K. Joseph M. Singh V. P. Singh A. K. (2008). Integrating marker assisted background analysis with foreground selection for identification of superior bacterial blight resistant recombinants in Basmati rice. Plant Breed. 127, 131–139. 10.1111/j.1439-0523.2007.01458.x

[B5] Guo J. Xu X. Li W. Zhu W. Zhu H. Liu Z.. (2016). Overcoming inter-subspecific hybrid sterility in rice by developing *indica*-compatible *japonica* lines. Sci. Rep. 6:26878. 10.1038/srep,26878 27246799 PMC4887987

[B6] ICAR-Indian Institute of Rice Research (2018). Progress Report 2017. Vol.1, Varietal Improvement, All India Coordinated Rice Improvement Project, ICAR-Indian Institute of Rice Research, Rajendranagar, Hyderabad – 500 030, T.S. Indiastat, Agriculture (2015-16). Available online at: http://www.indiastat.com/agriculture/2/stats.aspx

[B7] Kubo T. Yamagata Y. Eguchi M. Yoshimura A. (2008). A novel epistatic interaction at two loci causing male sterility in an inter-subspecific cross of rice (*Oryza sativa* L.). Genes Genet. Syst. 83, 443–453. 10.1266/ggs.83.443 19282622

[B8] Kubo T. Yoshimura A. (2000). Linkage analysis of a gene controlling F_2_ sterility in *japonica*/*indica* backcross progenies of rice. Rice Genet. Newslett. 15, 149–151.

[B9] Kumar S. Chakrabarti S. N. (2000). Genetic and cytogenetic analysis of spikelet sterility in *Indica* X *Japonica* crosses in rice (*Oryza sativa* L.). Indian J. Genet. Plant Breed. 60, 441–450.

[B10] Liu K. D. Zhou Z. Q. Xu C. G. Zhang Q. SaghaiMaroof M. A. (1996). An analysis of hybrid sterility in rice using a diallel cross of 21 parents involving *indica, japonica* and wide compatibility varieties. Euphytica 90, 275–280. 10.1007/BF00027476

[B11] Long Y. M. Zhao L. F. Niu B. X. Su J. Wu H. Chen Y. L.. (2008). Hybrid male sterility in rice controlled by interaction between divergent alleles of two adjacent genes. Proc. Nat. Acad. Sci. U.S.A. 105, 18871–18876. 10.1073/pnas.0810108105 19033192 PMC2596266

[B12] Mi J. Li G. Huang J. Yu H. Zhou F. Zhang Q. (2016). Stacking of S5-n and f-5n to overcome sterility in *indica*-*japonica* hybrid rice. Theor. Appl. Genet. 129, 563–575. 10.1007/s00122-015-2648-0 26704419

[B13] Ouyang Y. D. Chen J. J. Ding J. H. Zhang Q. F. (2009). Advances in the understanding of inter-subspecific hybrid sterility and wide compatibility in rice. Chin. Sci. Bull. 54, 2332–2341. 10.1007/s11434-009-0371-4

[B14] Pan X. B. Gu M. H. Chen Z. X. Hu X. Y. (1990). A comparative study on major wide compatibility varieties of rice. in Current Status of Two Line Hybrid Rice Research, ed Yuan L. P. (Beijing: Agricultural Press), 236–245.

[B15] Singh A. K. GopalaKrishana S. Singh V. P. Prabhu K. V. Mohapatra T. Singh N. K. (2011). Marker Assisted Selection: a paradigm shift in Basmati breeding. Indian J. Genet. Plant Breed. 71, 1–9.

[B16] Singh S. P. Sundaram R. M. Biradar S. K. Ahmed M. I. Viraktamath B. C. Siddiq E. A. (2006). Identification of simple sequence repeat markers for utilizing wide-compatibility genes in inter-subspecific hybrids in rice (*Oryza sativa* L.). Theor. Appl. Genet. 113, 509–517. 10.1007/s00122-006-0316-0 16788798

[B17] Sundaram R. M. Sakthivel K. Hariprasad A. S. Ramesha M. S. Viraktamath B. C. Neeraja C. N. (2010). Development and validation of a PCR-based functional marker system for the major wide-compatible gene locus *S5* in rice. Mol. Breed. 26, 719–727. 10.1007/s11032-010-9482-5

[B18] Van Berloo R. (1999). GGT: Software for display of graphical genotypes. J. Hered. 90, 328–329. 10.1093/jhered/90.2.328

[B19] Vijaya Kumar C. H. M. Ilyas Ahmed M. Viraktamath B. C. Ramesha M. S. (1999). Identification and utilization of wide compatibility gene in rice. Indian J. Genet. 59, 139–148.

[B20] Vijayakumar R. Virmani S. S. (1992). Wide compatibility in rice (*Oryza sativa* L.). Euphytica 64, 71–80.

[B21] Virmani S. S. (1996). Hybrid Rice. Adv. Agro. 57, 377–462. 10.1016/S0065-2113(08)60928-1

[B22] Virmani S. S. Virakamath B. C. Laral C. L. Toledo R. S. Lopez M. T. Manalo J. O. (1997). Hybrid Rice Breeding Manual, Vol. 151 Los Baños: Philippines IRRI.

[B23] Wang J. Liu K. D. Xu C. G. Li X. H. Zhang Q. (1998). The high level of wide compatibility of variety ‘Dular’ has a complex genetic basis. Theor. Appl. Genet. 97, 407–411. 10.1007/s001220050910

[B24] Yang Y. X. Li Y. H. Tong J. F. Shahid M. Q. Chen Z. X. Wang L. (2012). Wide-compatibility gene *S5*^*n*^ exploited by functional molecular markers and its effect on fertility of intersubspecific rice hybrids. Crop Sci. 52, 669–675. 10.2135/cropsci2011.04.0232

[B25] Yuan L. P. (1994). Increasing yield potentials in rice by exploitation of heterosis, in Hybrid Rice Technology: New Development and Future Prospects, ed Virmani S. S. (Manila: IRRI), 1–6.

[B26] Zhang Q. Liu K. D. Yang G. P. SaghaiMaroof M. A. Xu C. G. Zhou Z. Q. (1997). Molecular marker diversity and hybrid sterility in *indica*-*japonica* rice crosses. Theor. Appl. Genet. 95, 112–118. 10.1007/s001220050538

