# Supplementary material for: Marker-Assisted Improvement of the Elite Maintainer Line of Rice, IR 58025B for Wide Compatibility (S5n) Gene
Source: Front Plant Sci. 2018 Jul 20;9:1051. doi: 10.3389/fpls.2018.01051 (PMC6062963; doi:10.3389/fpls.2018.01051)
Supplement: Supplementary file 1 [file Table_1.DOCX]

**SUPPLEMENTARY TABLE 1 I** Gene based functional marker for foreground selection of the wide compatibility gene (*S5^n^*)

| **Trait** | **Gene** | **Marker** | **Chr.** |  | **Primer Sequence** | **AT** | | **Reference** |
| --- | --- | --- | --- | --- | --- | --- | --- | --- |
|  |  |  |  |  |  | **(°C)** | |  |
| Wide compatibility | *S5^n^* | S5-InDel | 6 |  | **F**-CCTACGTTTGACTGCCTGCCTG  **R**-CTACACGCGGCTTCGGGAAAGC | 58 | | Sundaram et al. 2010 |
| Aroma | *badh2* | *nksbadh2* | 8 |  | **F**-GGTTGCATTTACTGGGAGTTATG  **R**-TCCACAGAAATTTGGAAACAAAC | | 58 | Singh et al. 2011 |
| **Chr. :** Chromosome**; MD:AT:** Annealing Temperature**; F:** Forward; **R:** Reverse | | | | | | | | |
