# Supplementary material for: Marker-Assisted Improvement of the Elite Maintainer Line of Rice, IR 58025B for Wide Compatibility (S5n) Gene
Source: Front Plant Sci. 2018 Jul 20;9:1051. doi: 10.3389/fpls.2018.01051 (PMC6062963; doi:10.3389/fpls.2018.01051)
Supplement: Supplementary file 2 [file Table_2.DOCX]

**SUPPLEMENTARY TABLE 2 I** List of polymorphic microsatellite markers identified between IR58025B and Dular

| Chr. No. | Number of marker used | Polymorphic markers identified | % Polymorphism |
| --- | --- | --- | --- |
| 1 | 56 | 28; RM3252 (0.30), RM3148* (0.74), RM1247 (1.65), RM8068 (1.65), RM1* (4.63), RM7466* (5.78), RM1360 (6.21), RM490* (6.67), RM5800* (7.39), RM576 (8.13), RM600 (9.46), RM8071 (9.48), RM493* (12.28), RM306 (21.58), RM9* (23.32), RM5 (23.97), RM2318 (24.14), RM488 (24.80), RM443* (28.33), RM11573* (29.32), RM1152 (30.09), RM11629 (30.47), RM11722 (32.44), RM212 (33.05), RM529 (40.67), RM5794 (41.08), RM5536* (41.16), RM12276 (43.04) | 50.00 |
| 2 | 51 | 28; RM6842 (0.12), RM7382* (0.12), RM7252 (0.13), RM3340 (0.38), RM12363 (1.19), RM12368 (1.27), RM12375 (1.41), RM6616 (2.86), RM279* (2.88), RM12492 (3.16), RM4355 (4.26), RM12673 (5.67), RM6375* (9.55), RM452 (9.56), RM12939* (9.86), RM290 (10.80), RM5812 (15.89), RM561 (18.76), RM341* (19.34), RM106 (25.14), RM3763 (27.92), RM573* (27.94), RM6 (29.57), RM318 (29.63), RM240 (31.49), RM13971 (31.55), RM1063 (35.09), RM7485* (35.73) | 54.90 |
| 3 | 41 | 23; RM14234* (0.02), RM3654 (0.08), RM3265 (0.10), RM3202 (0.80), RM14302 (0.86), RM22 (1.51), RM569 (1.90), RM5474 (3.80), RM3392 (3.82), RM489 (4.33), RM7576 (6.05), RM7* (9.82), RM14898 (12.43), RM15281* (18.68), RM15573 (25.01), RM15741* (27.93), RM3525 (30.38), RM416 (31.24), RM468 (32.67), RM7000* (33.74), RM227 (34.92), RM514 (35.28), RM7389* (36.15) | 56.10 |
| 4 | 38 | 28; RM551* (0.17), RM335 (0.68), RM16335 (1.70), RM261* (6.55), RM307* (13.14), RM16792 (18.01), RM16830* (18.68), RM1359 (19.86), RM3708 (20.65), RM119 (21.24), RM3042 (22.83), RM3866* (23.17), RM6540* (23.44), RM273 (23.86), RM252 (25.17), RM241(26.85), RM17377* (28.93), RM17479 (30.90), RM17506 (31.52), RM17521 (31.86), RM8217 (32.65), RM17604 (33.64), RM3466 (33.86), RM131 (34.42), RM280 (34.98), RM559* (35.15), RM5608 (35.17), RM3531 (35.21) | 73.68 |
| 5 | 49 | 26; RM122* (0.31), RM17863 (2.18), RM413 (2.21), RM592 (2.79), RM13 (2.99), RM17959* (3.84), RM17960 (3.84), RM17962 (3.86), RM437 (3.87), RM3381* (9.58), RM249* (10.77), RM18452* (16.32), RM598 (16.75), RM459* (20.17),eSSR1(23.64), RM3476 (23.84), RM5970 (23.94), RM7081(24.52), RM7446 (24.95), RM178* (25.10), RM3620 (25.20), RM3790 (26.19), RM3170* (27.93), RM31 (28.61), RM5907 (29.54), RM19218* (29.60) | 53.06 |
| 6 | 53 | 31; RM6775* (0.20), RM7158 (0.21), RM508 (0.44), RM1369 (1.56), RM588 (1.61), RM8125 (2.69), RM8200 (2.69), RM204 (3.16), RM225* (3.41), RM19454 (3.94), RM19462 (4.00), RM19483 (4.22), RM217* (4.23), RM19521 (4.71), RM19545 (4.99), RM19552 (5.21), RM19555 (5.23), RM253* (5.42), S5-InDel (5.75), BF-S5 (5.75), S5-t1 (5.75), RM276* (6.23), RM136* (8.75), RM20069* (16.54), RM6818 (16.58), RM20352* (24.00), RM528 (26.55), RM340* (28.59), RM3343* (29.61), RM3765 (29.72), RM5463* (30.98) | 58.49 |

***** Denotes markers used for background selection, values presented inparenthesis represent physical position of the marker in Mb.

**Supplementary Table 2** Contd.

| Chr. No. | Number of marker used | Polymorphic markers identified | % Polymorphism |
| --- | --- | --- | --- |
| 5 | 49 | 26; RM122* (0.31), RM17863 (2.18), RM413 (2.21), RM592 (2.79), RM13 (2.99), RM17959* (3.84), RM17960 (3.84), RM17962 (3.86), RM437 (3.87), RM3381* (9.58), RM249* (10.77), RM18452* (16.32), RM598 (16.75), RM459* (20.17),eSSR1(23.64), RM3476 (23.84), RM5970 (23.94), RM7081(24.52), RM7446 (24.95), RM178* (25.10), RM3620 (25.20), RM3790 (26.19), RM3170* (27.93), RM31 (28.61), RM5907 (29.54), RM19218* (29.60) | 53.06 |
| 6 | 53 | 31; RM6775* (0.20), RM7158 (0.21), RM508 (0.44), RM1369 (1.56), RM588 (1.61), RM8125 (2.69), RM8200 (2.69), RM204 (3.16), RM225* (3.41), RM19454 (3.94), RM19462 (4.00), RM19483 (4.22), RM217* (4.23), RM19521 (4.71), RM19545 (4.99), RM19552 (5.21), RM19555 (5.23), RM253* (5.42), S5-InDel (5.75), BF-S5 (5.75), S5-t1 (5.75), RM276* (6.23), RM136* (8.75), RM20069* (16.54), RM6818 (16.58), RM20352* (24.00), RM528 (26.55), RM340* (28.59), RM3343* (29.61), RM3765 (29.72), RM5463* (30.98) | 58.49 |
| 7 | 41 | 16; RM20827* (0.52), RM20884 (1.30), RM298 (2.76), RM180* (5.73), RM3859* (8.87), RM5543 (15.75), RM5875* (15.99), RM432* (18.95), RM336 (21.87), RM6403 (22.17), RM10 (22.18), RM473 (25.45), RM234* (25.47), RM22081 (27.65), RM22105 (28.08), RM248* (29.33) | 39.02 |
| 8 | 33 | 19; RM337* (0.15), RM310 (5.11), RM22529* (5.18), RM44* (11.75), RM404 (15.43), RM23001 (17.71), RM339 (17.94), RM23077* (19.64), nksbadh2 (20.38), RM223 (20.65), RM23174 (21.07), RM210 (22.47), RM3845* (24.75), RM3452 (24.77), RM419 (25.82), RM230 (25.83), RM3480 (27.26), RM6948 (27.31), RM281* (27.89) | 57.58 |
| 9 | 30 | 12; RM23654* (0.15), RM23742* (2.71), RM5526 (7.31), RM23928 (7.51), RM23958* (7.99), RM410 (17.64),RM257* (17.71), RM3249* (19.74), RM215 (21.18), RM24718 (21.20), RM6797 (22.71), RM205* (22.72) | 40.00 |
| 10 | 30 | 15; RM6364* (0.06), RM6461(0.17), RM7361 (1.92), RM3882 (2.73), RM216* (5.10), RM239* (9.69), RM294A (10.07), RM5689* (13.48), RM269* (18.02), RM6737 (18.71), RM25653 (18.80), RM6100* (18.81), RM25664* (19.04), RM1108* (19.16), RM228* (22.24) | 50.00 |
| 11 | 33 | 16; RM286 (0.38), RM7203* (1.08), RM26063 (2.25), RM7557 (2.34), RM2459 (2.40), RM202* (9.00), RM26406 (9.05), RM6091 (13.40), RM3428 (13.48), RM7226* (14.05), RM26632 (14.70), RM287 (16.76), RM21* (19.17), RM206* (22.01), RM254 (23.76), RM224* (27.20) | 48.48 |
| 12 | 31 | 16; RM5568* (0.71), RM7315 (2.18), RM3747 (2.30), RM247* (3.18), RM101 (8.82), RM27877* (9.17), RM7102* (13.21), RM28074 (14.94), RM1246* (19.08), RM28283 (19.49), RM6022 (19.88), RM28585 (24.38), RM28607 (24.76), RM270 (24.96), RM1159* (25.90), RM1300 (25.96) | 51.61 |
| Total | **486** | **258** | **53.09** |

***** Denotes markers used for background selection, values presented in parenthesis represent physical position of the marker in Mb.
