# Supplementary material for: Marker-Assisted Improvement of the Elite Maintainer Line of Rice, IR 58025B for Wide Compatibility (S5n) Gene
Source: Front Plant Sci. 2018 Jul 20;9:1051. doi: 10.3389/fpls.2018.01051 (PMC6062963; doi:10.3389/fpls.2018.01051)
Supplement: Supplementary file 3 [file Table_3.DOCX]

**SUPPLEMENTARY TABLE 3 I** Number of backcross plants screened at each generation and number of plants possessing *S5^n^*^++^/*S5^n^*^+-^ and *badh2*^++^/*badh2*^+-^ genes and the % recovery of IR 58025B in the selected backcross plants

| S. No | Generation | No. of plants screened | No. of plants *S5^n^*^++^/*S5^n^*^+-^gene | No. of plants possessing *S5^n^*^++^/*S5^n^*^+-^ and*badh2*^++^/*badh2*^+-^genes | No. of plant selected possessing*S5^n^*^++^/*S5^n^*^+-^and*badh2*^++^/*badh2*^+-^genes | RGA% |
| --- | --- | --- | --- | --- | --- | --- |
| 1 | BC_1_F_1_ | 960 | 470 | 230 | 14 | 70.32-74.72^#^ |
| 2 | BC_2_F_1_ | 250 | 126 | 60 | 14 | 80.21-86.81^#^ |
| 3 | BC_3_F_1_ | 240 | 116 | 50 | 10 | 88.46-92.30^#^ |
| 4 | BC_3_F_2_ | 800 | 184 | 46 | 20 | 93.10-94.50^#^ |
| 5 | BC_3_F_3_ | 20 | 20 | 20 | 10 | 93.85-96.15^#^ |
| 6 | BC_3_F_4_ | 10 | 10 | 10 | 10 | 94.51-98.90^#^ |

^# %^recurrent parent genome recovery (RGA %) was calculated at each generation by dividing sum of the number of rice parental polymorphic SSR markers which are homozygous for the recurrent specific allele and half of heterozygous allele and with the total number of parental polymorphic SSR markers (#91)
