# Supplementary figures and images for: Marker-Assisted Improvement of the Elite Maintainer Line of Rice, IR 58025B for Wide Compatibility (S5n) Gene
Source: Front Plant Sci. 2018 Jul 20;9:1051. doi: 10.3389/fpls.2018.01051 (PMC6062963; doi:10.3389/fpls.2018.01051)

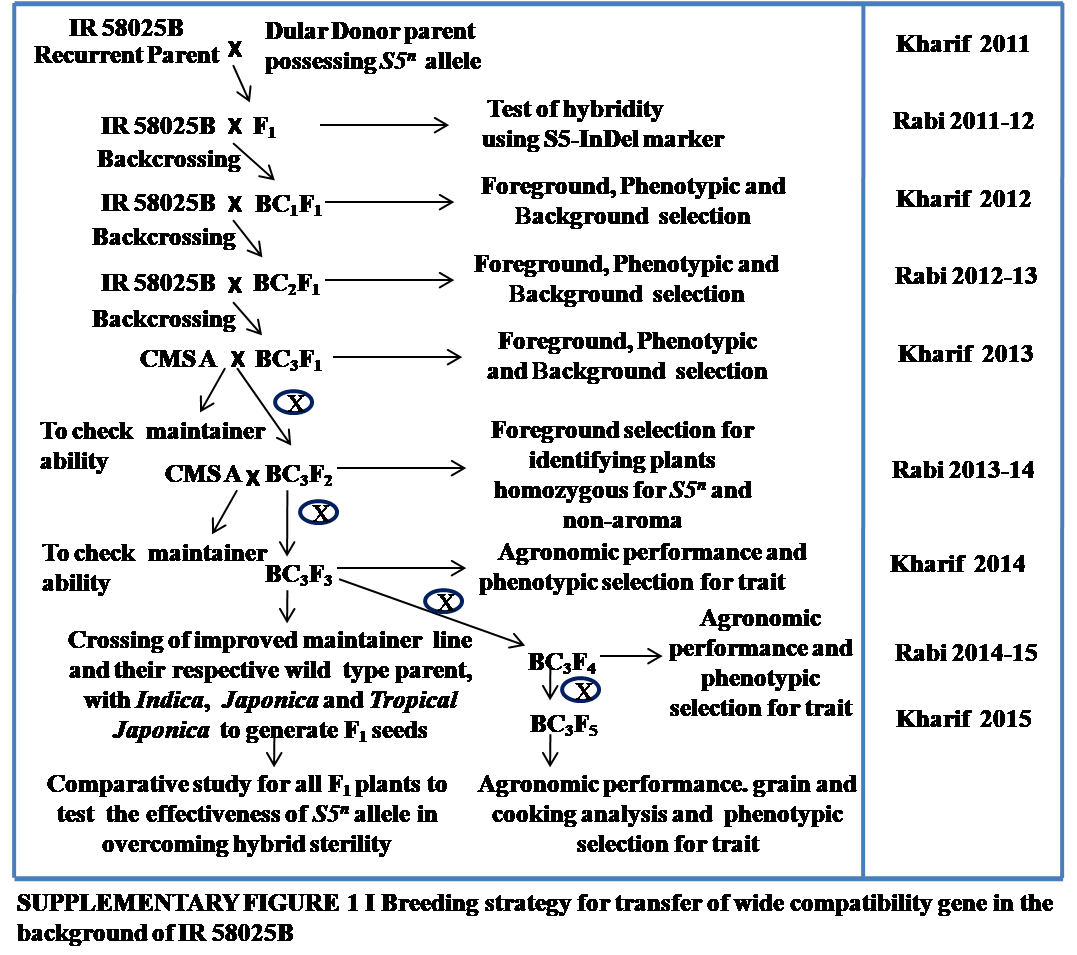

Supplement: Supplementary file 4 [file Image_1.TIF]

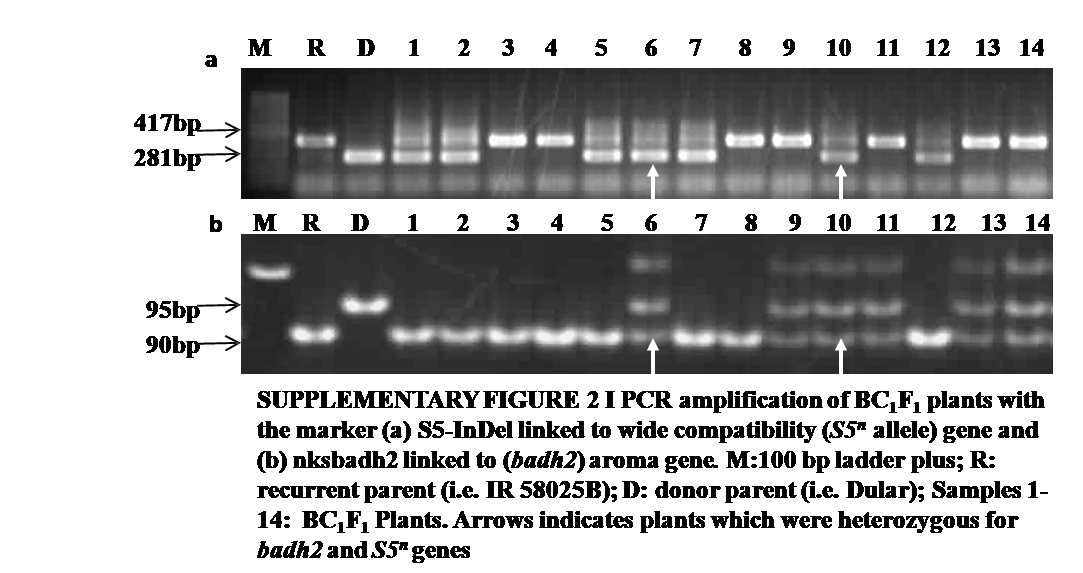

Supplement: Supplementary file 5 [file Image_2.TIF]
